# Antifungal Activity of Naphthoquinoidal Compounds *In Vitro* against Fluconazole-Resistant Strains of Different *Candida* Species: A Special Emphasis on Mechanisms of Action on *Candida tropicalis*

**DOI:** 10.1371/journal.pone.0093698

**Published:** 2014-05-09

**Authors:** João B. A. Neto, Cecília R. da Silva, Maria A. S. Neta, Rosana S. Campos, Janaína T. Siebra, Rose A. C. Silva, Danielle M. Gaspar, Hemerson I. F. Magalhães, Manoel O. de Moraes, Marina D. P. Lobo, Thalles B. Grangeiro, Tatiane S. C. Carvalho, Emilay B. T. Diogo, Eufrânio N. da Silva Júnior, Felipe A. R. Rodrigues, Bruno C. Cavalcanti, Hélio V. N. Júnior

**Affiliations:** 1 Department of Clinical and Toxicological Analysis, School of Pharmacy, Laboratory of Bioprospection and Experiments in Yeast (LABEL), Federal University of Ceará, Fortaleza, Ceará, Brazil; 2 Department of Pathology and Legal Medicine, School of Medicine, Laboratory of Bioprospection and Experiments in Yeast (LABEL), Federal University of Ceará, Fortaleza, Ceará, Brazil; 3 Department of Pharmaceutical Sciences, Center for Toxicological Assistance, University Federal of Paraíba, Paraíba, Brazil; 4 Department of Physiology and Pharmacology, Laboratory of Experimental Oncology, Federal University of Ceará, Fortaleza, Ceará, Brazil; 5 Department of Biology, ScienceCenter, Molecular Genetics Laboratory, Federal University of Ceará, Ceará, Brazil; 6 Natural Products Research Nucleus, Federal University of Rio de Janeiro, Rio de Janeiro, Brazil; 7 Department of Chemistry, Institute of Exact Sciences, Laboratory of Synthetic and Heterocyclic Chemistry, Federal University of Minas Gerais, Minas Gerais, Brazil; Universidade de Sao Paulo, Brazil

## Abstract

In recent decades, the incidence of candidemia in tertiary hospitals worldwide has substantially increased. These infections are a major cause of morbidity and mortality; in addition, they prolong hospital stays and raise the costs associated with treatment. Studies have reported a significant increase in infections by non-*albicans Candida* species, especially *C. tropicalis*. The number of antifungal drugs on the market is small in comparison to the number of antibacterial agents available. The limited number of treatment options, coupled with the increasing frequency of cross-resistance, makes it necessary to develop new therapeutic strategies. The objective of this study was to evaluate and compare the antifungal activities of three semisynthetic naphthofuranquinone molecules against fluconazole-resistant *Candida* spp. strains. These results allowed to us to evaluate the antifungal effects of three naphthofuranquinones on fluconazole-resistant *C. tropicalis*. The toxicity of these compounds was manifested as increased intracellular ROS, which resulted in membrane damage and changes in cell size/granularity, mitochondrial membrane depolarization, and DNA damage (including oxidation and strand breakage). In conclusion, the tested naphthofuranquinones (compounds 1–3) exhibited *in vitro* cytotoxicity against fluconazole-resistant *Candida* spp. strains.

## Introduction

The incidence of candidemia in tertiary hospitals worldwide has substantially increased in recent decades. Candidemia is a major cause of morbidity and mortality, especially in immune-compromised patients and those who are hospitalized with severe underlying diseases. Moreover, these infections prolong hospital stays and increase the costs associated with treatment. Therefore, *Candida* species represent a group of medically important pathogenic fungi [1]. Previous studies have reported a significant increase in the number of infections by non-*albicans* species (*C. tropicalis*, *C. glabrata*, *C. parapsilosis* and *C. krusei*), corresponding to 36–63% of all cases [2]. As an invasive fungus, *Candida tropicalis* is a major non-*albicans Candida* species that causes fungemia in patients [3], and is the second-most commonly isolated *Candida* species in Brazilian hospitals [4]. The widespread occurrence of *C. tropicalis* in Brazilian hospitals may be explained by the high level of resistance to certain antifungal drugs among non-*albicans* species, as demonstrated by the numerous reports of fluconazole-resistant clinical isolates of *C. tropicalis* in recent years [5].

Antimicrobial resistance has become increasingly important in antifungal therapy because resistance to almost all antifungal agents, especially fluconazole, has been found in clinical isolates of *Candida* spp. This resistance constitutes a major public health problem because there are a limited number of antifungal agents available on the market [6]. Thus, the search for new therapeutic strategies has become a pressing concern [7].

Quinones are a large and varied family of organic metabolites from natural sources with chemical properties that allow them to interact with biological targets. Interest in these substances has intensified in recent years due to their critical importance in several biochemical processes, and quinones have become increasingly prominent in pharmacological studies [8]. Naphthoquinones are an important quinone subclass of biologically active molecules that possess antibacterial, antifungal, antiviral, anti-inflammatory, antipyretic, anticancer and trypanocidal activities [9], [10].

Natural products are an important source of candidates for anti-infective and antitumor agents. However, natural products have problems related to their physicochemical characteristics, implying deficient pharmacokinetic parameters. Moreover, obtaining large amounts of a product of natural origin precludes its use as a drug. As a result, semi-synthesis has emerged as a tool for further structural modification in order to modulate the biological properties of these purely natural products (the naphthoquinones for instance) [11]. The clinical importance of these molecules stimulated the search and synthesis of new agents, because naphthoquinones derivative substitutes may have a more pronounced antifungal activity [12].

**Figure 1:**
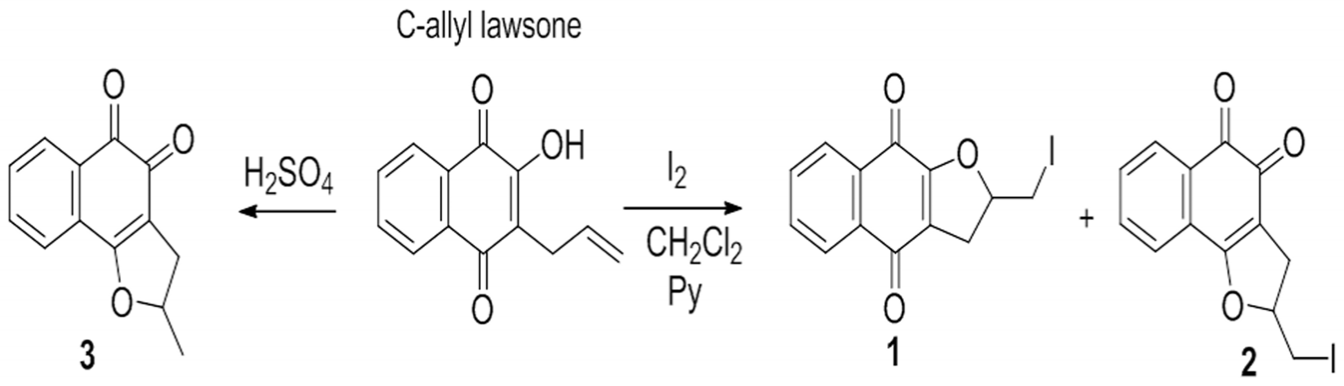
The synthesis of compounds 1, 2 and 3.

The aim of the current study was to evaluate the antifungal activity of methylated and iodinated naphthofuranquinones obtained from C-allyl lawsone (Figure 1) [13] against isolates of fluconazole-resistant *Candida* spp.

## Results

### Molecular identification

The complete ITS/5.8S region (ITS1, 5.8S, and ITS2) of the nuclear ribosomal DNA from the isolates used in the present study was amplified, sequenced and compared to the sequences deposited in the GenBank database. The BLAST searches revealed that the sequences from nine, two and three isolates were identical to the ITS/5.8S sequences from different isolates and strains of *C. tropicalis*, *C. parapsilosis* and *C. albicans*, respectively. Therefore, the isolates were identified as belonging to these species (Table 1).

**Table 1:** The effects of naphthoquinoidal compounds (NFQs) 1, 2 and 3 against FLC-resistant strains of *Candida* spp. isolated in Ceará (MIC 50% at 24 h).

			MIC		
			Standard MIC		
Strains^a^	Origin	FLC b(µg/mL)	1 (µg/mL)	2 (µg/mL)	3 (µg/mL)
*C.tropicalis 1*	Blood	≥8.0	25.40	25.40	25.40
*C.tropicalis 2*	Blood	≥8.0	32	25.40	20.15
*C.tropicalis 3*	Blood	≥8.0	20.15	32	10.10
*C.tropicalis 4*	Blood	≥8.0	25.40	25.40	16
*C.tropicalis 5*	Blood	≥8.0	40.32	25.40	32
*C.tropicalis 6*	Blood	≥8.0	64	40.32	40.32
*C.tropicalis 7*	Blood	≥8.0	64	40.32	40.32
*C.tropicalis 8*	Blood	≥8.0	37.3	42.6	18.7
*C.tropicalis 9*	Urine	≥8.0	42.6	37.3	37.3
*C.parapsilosis 1*	Blood	≥8.0	16	16	16
*C.parapsilosis 2*	CSF	≥8.0	16	64	16
*C.albicans 1*	Blood	≥8.0	10.7	8	8
*C.albicans 2*	Blood	≥8.0	12	96	16
*C.albicans 3*	Blood	≥8.0	16	96	12

a FLC-resistant strains of *Candida* spp. isolated from biological samples.

b FLC – fluconazole; compounds (1-3) – NFQs. The MIC was defined as the lowest concentration that produced a 50% reduction in the growth of fungal cells after 24 h of incubation. The microdilution in broth was performed according to CLSI protocol M27-A3. The FLC concentrations ranged from 0.125–64 µg/mL and the compound (1-3) concentrations varied from 0.25–128 µg/mL. The MICs represent the geometric means of at least three MICs determined on different days.

### Antifungal effects of naphthofuranquinones against fluconazole-resistant *C. tropicalis*

The susceptibility of each strain to fluconazole was evaluated using microdilutions. After 24 h of exposure to fluconazole, all of the tested *C. tropicalis, C.parapsilosis e C.albicans* strains were characterized as resistant to fluconazole, with MIC values most equal 8 µg/mL. The growth inhibition induced by the different naphthoquinoidal compounds was also determined. Compounds 1-3 displayed antifungal activities (Table 1).

### Changes in cell size/granularity, loss of cell viability and plasma membrane damage in fluconazole-resistant *C. tropicalis* after treatment with naphthofuranquinones

**Figure 2:**
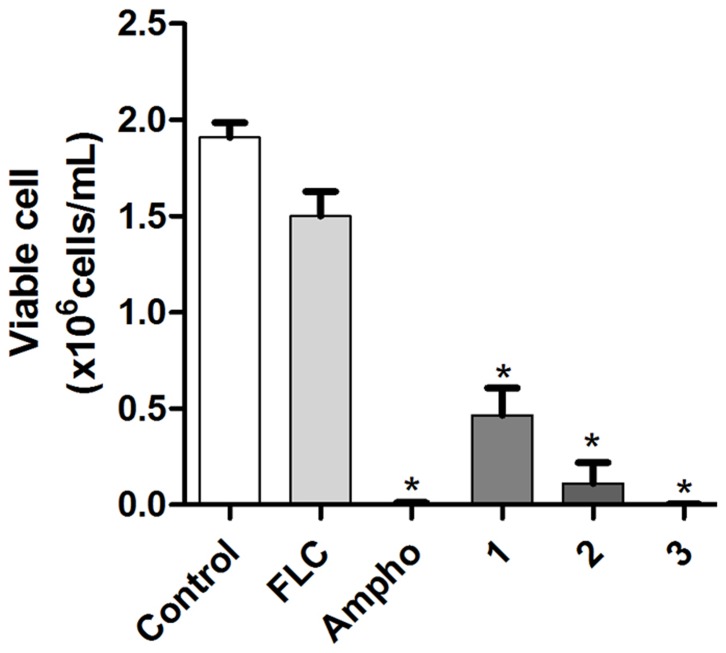
A flow cytometric analysis of the reduction in cell number to reveal the antifungal effect of naphthofuranquinone (NFQ) compounds 1, 2 and 3 at concentrations of 32 µg/mL (a); 64 µg/mL (b) and 128 µg/mL (c) on isolates of FLC-resistant *C. tropicalis* after 24 h incubation. * P<0.05 compared with the control using an ANOVA followed by the Newman-Keuls test.

**Figure 3:**
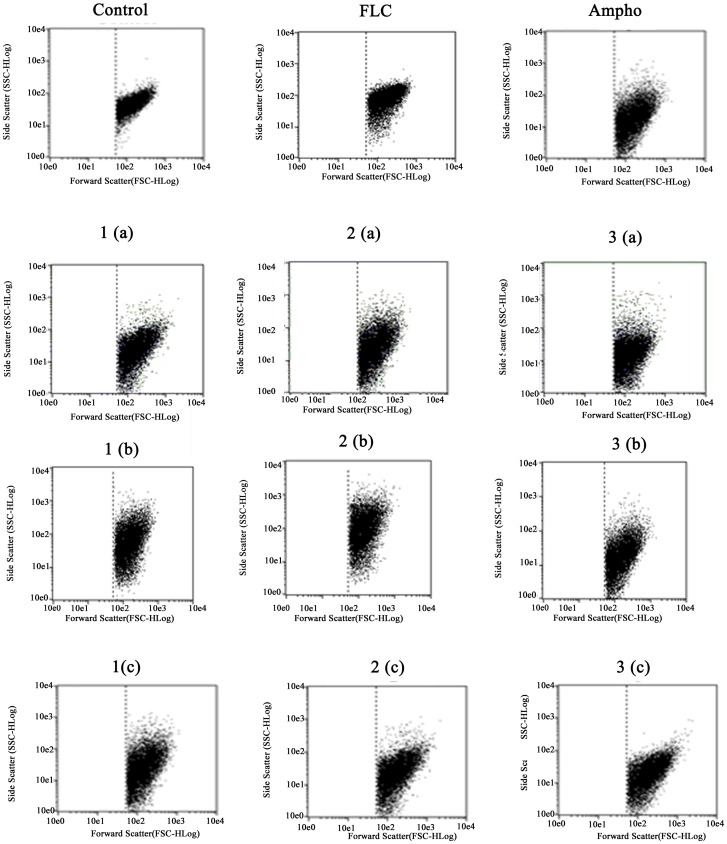
An analysis of changes in cell size/granularity (FSC×SSC) to evaluate the effects of fluconazole (64 µg/mL), Ampho (4 µg/mL) and naphthofuranquinone (NFQ) compounds 1, 2 and 3 at concentrations of 32 µg/mL (a); 64 µg/mL (b) and 128 µg/mL (c) on isolates of FLC-resistant *C. tropicalis* after a 24 h incubation. * p<0.05 compared to the control using an ANOVA followed by the Newman-Keuls test.

**Figure 4:**
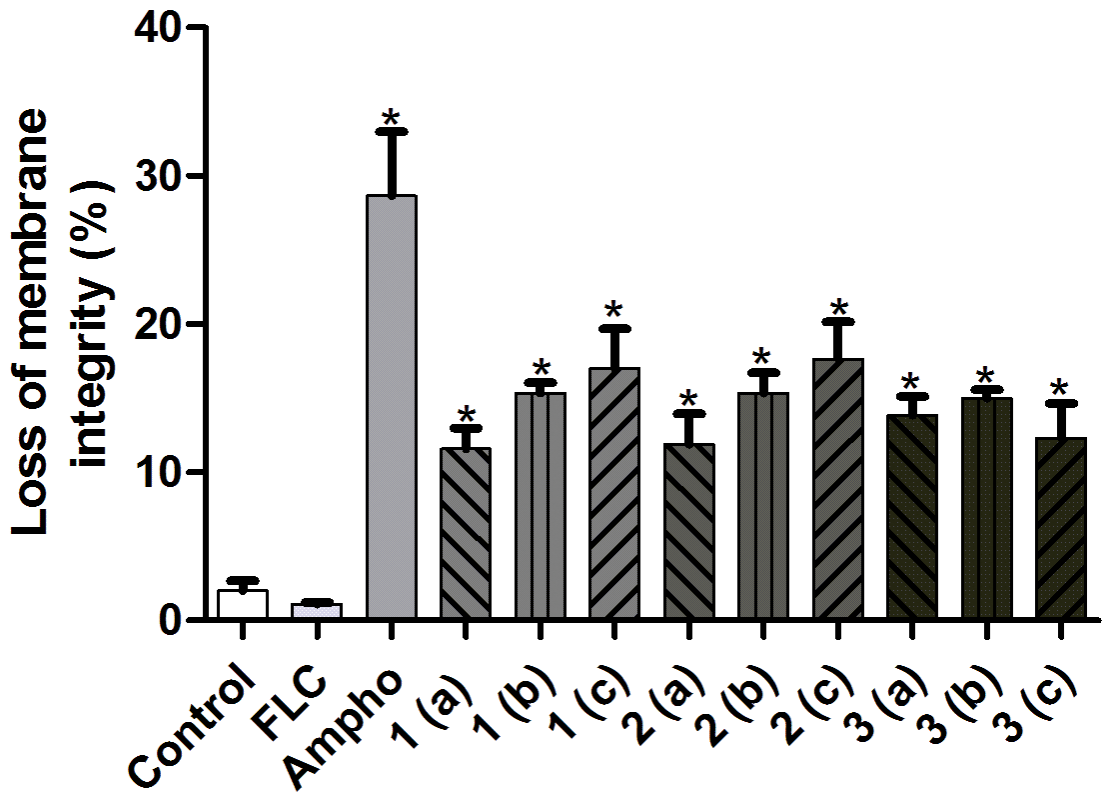
Effects of fluconazole (64 µg/mL), Ampho (4 µg/mL) and naphthofuranquinone (NFQ) compounds 1, 2 and 3 at concentrations of 32 µg/mL (a); 64 µg/mL (b) and 128 µg/mL (c) on membrane integrity (as determined by a PI exclusion test) on isolates of FLC-resistant *C. tropicalis* after 24 h incubation. The population of cells in each lower right quadrant corresponds to the percentage of cells with damaged membranes (PI positive). * p<0.05 compared to the control using an ANOVA followed by the Newman-Keuls test.

As shown in Figure 2, there was no significant reduction in the number of viable cells when the fluconazole-resistant strains were treated with the azole, in comparison to untreated cells (P<0.05). In contrast to this, when the fluconazole-resistant cells were treated with the naphthofuranquinones (compounds 1-3) for 24 h they exhibited significant decreases in cell viability (P<0.05). Changes in cell size/granularity were observed when the fluconazole-resistant *C. tropicalis* strains were exposed to the naphthofuranquinone compounds 1-3 for 24 h. evaluated, the above changes in cell size/granularity were observed only after 24 h of exposure to naphthofuranquinone compounds 1-3 (Figure 3). Moreover, as shown in Figure 3, the fluconazole-resistant strains did not display any significant increase in plasma membrane instability after exposure to fluconazole. However, the treatment of these fluconazole-resistant strains with the naphthofuranquinones for 24 h induced a significant increase (P<0.05) in the number of cells with plasma membrane lesions (Figure 4).

### Increased intracellular levels of ROS in *C. tropicalis* after exposure to naphthofuranquinones

**Figure 5:**
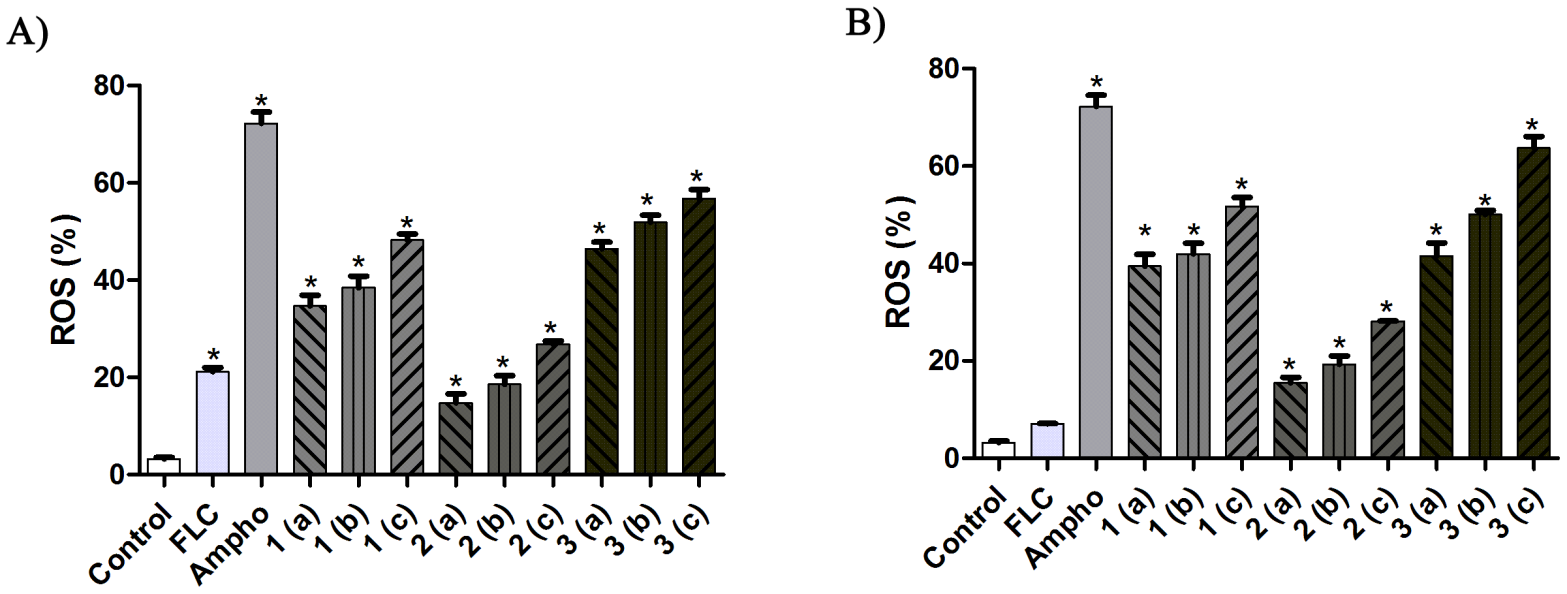
An evaluation of ROS formation in fluconazole-sensitive (A) and fluconazole-resistant (B) *C. tropicalis* isolates after treatment with naphthofuranquinone (NFQ) compounds 1, 2 and 3 using the concentrations 32 µg/mL (a); 64 µg/mL (b) and 128 µg/mL (c). The percentage of ROS formation in the fluconazole-sensitive and fluconazole-resistant *C. tropicalis* isolates was evaluated for 24 hours. * P<0.05 compared with the control using an ANOVA followed by the Newman-Keuls test.

The cells of *C. tropicalis* sensitive and resistant to fluconazole when untreated (control) showed no significant differences in the levels of intracellular ROS. However, C. *tropicalis* cells from strains that were both sensitive and resistant to fluconazole showed significant increases (P<0.05) in ROS production after exposure to compounds 1-3 compared with the negative control group (Figure 5).

### Changes in the mitochondrial transmembrane potential (Δψm)

**Figure 6:**
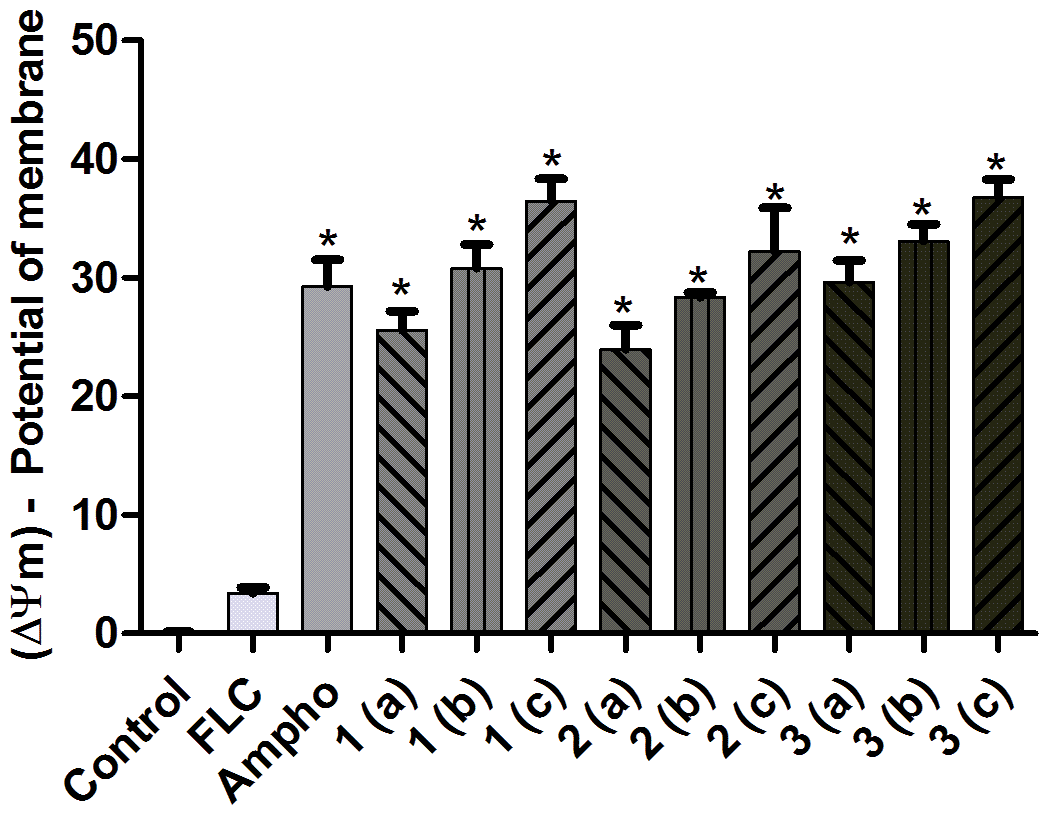
An assessment of the mitochondrial membrane potential (Δψm) of fluconazole-resistant *C. tropicalis* strains. The cells were labeled with Rh123 (50 nM). The graph shows strains incubated for 24 hours with RPMI-1640 (control), with FLC (64 µg/mL) and Ampho (4 µg/mL) and with naphthofuranquinone (NFQ) compounds 1, 2 and 3 at concentrations of 32 µg/mL (a); 64 µg/mL (b) and 128 µg/mL (c) * P<0.05 compared with the control using an ANOVA followed by the Newman-Keuls test.

After 24 h of exposure, significant changes (P<0.05) in the Δψm were observed in fluconazole-resistant *C. tropicalis* strains treated with naphthofuranquinones (Figure 6) in comparison to untreated cells (Figure 6). Amphotericin B was used as a positive control.

### DNA Damage

**Figure 7:**
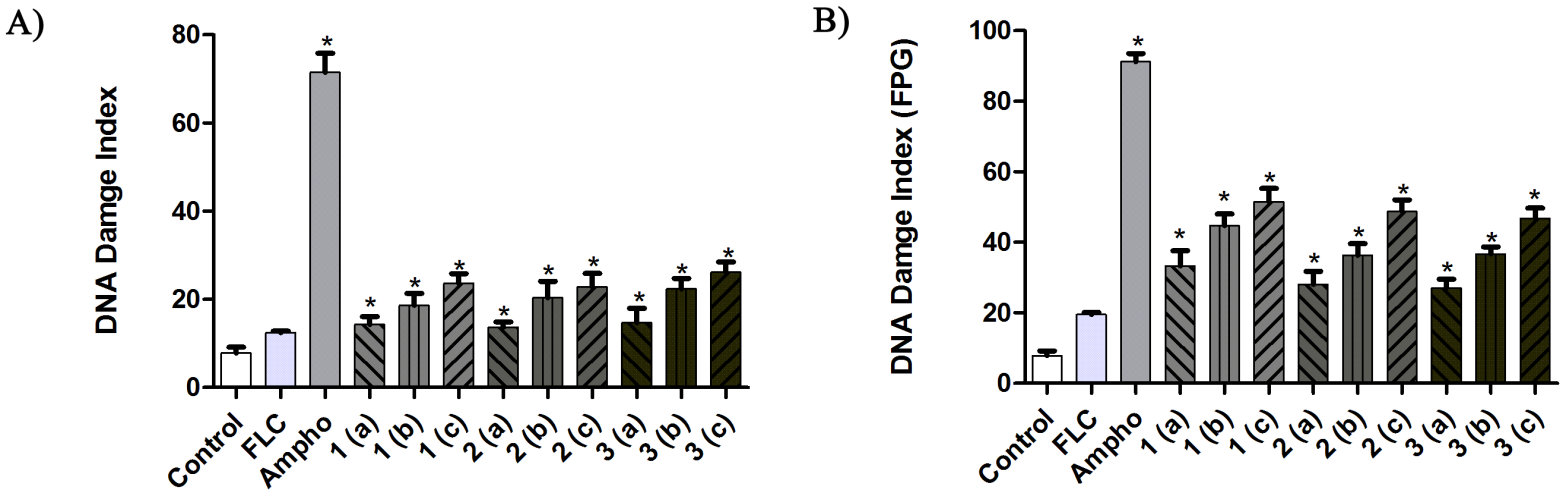
The effects of 24-1640 (control), with FLC (64 µg/mL), Ampho (4 µg/mL), and naphthofuranquinone (NFQ) compounds 1, 2 and 3 at concentrations of 32 µg/mL (a); 64 µg/mL (b) and 128 µg/mL (c) on the DNA damage index and modified alkaline versions (FPG) of the comet assay were used in FLC-resistant strains of *C. tropicalis*. * P<0.05 compared with the control using an ANOVA followed by the Newman-Keuls test.

As shown in Figure 7, the three tested naphthofuranquinone compounds induced a significant (P<0.05) increase in DNA damage in the fluconazole-resistant *C. tropicalis* strains in comparison to untreated cells (P<0.05). Furthermore, the fluconazole-resistant strains that had been incubated with FPG also showed a significant increase in the DI values after the treatment with naphthofuranquinones in comparison to the control group (P<0.05). Amphotericin B was used as a positive control.

### Phosphatidylserine externalization in *C. tropicalis*

**Figure 8:**
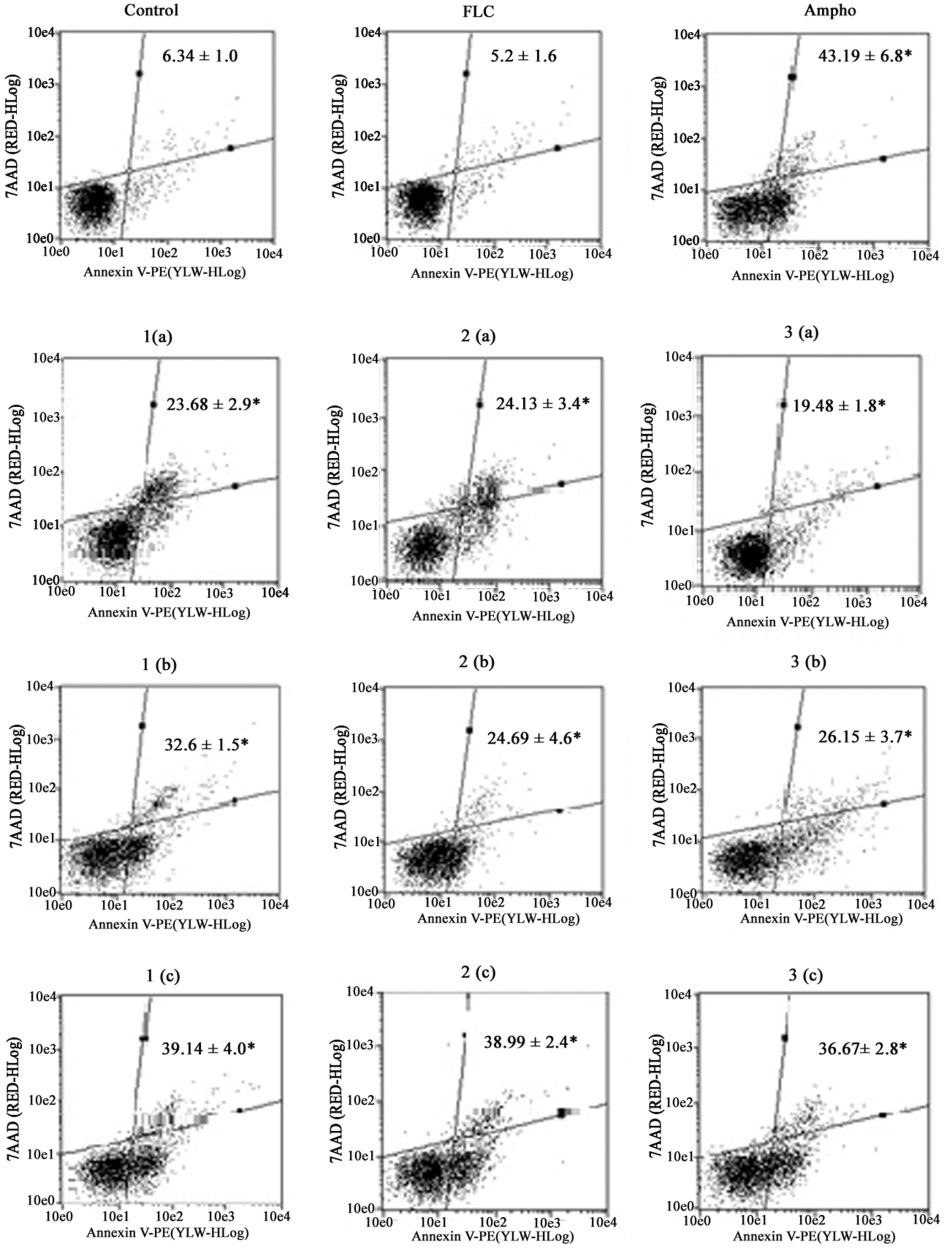
Phosphatidylserine externalization, which is observed at an early stage of apoptosis, was shown by annexin V staining. This probe enabled us to detect alterations of phosphatidylserine from the inner membrane to outer membrane. The intensity of fluorescence means the quantity of exposed phosphatidylserine cells treated with FLC (64 µg/mL), with Ampho (4 µg/mL) and naphthofuranquinone (NFQ) compounds 1, 2 and 3 at concentrations of 32 µg/mL (a); 64 µg/mL (b) and 128 µg/mL (c) for 24 hours. *P<0.05 compared to control by ANOVA followed by Newman Keuls test.

In Figure 8, the populations of cells in the lower and upper right quadrants, respectively, correspond to early (Annexin V-positive, 7AAD-negative) and late (Annexin V-positive, 7AAD-positive) apoptotic cells with externalized phosphatidylserine. After 24 h exposure, the percentage of all cells (in the early and late apoptotic stages) with externalized phosphatidylserine after a single treatment with naphthofuranquinone compounds showed a significant increase (P<0.05) in comparison to untreated cells. The treatment of fluconazole-resistant cells with the compounds 1-3 clearly induced cell death in a similar way to what was found in cells treated with amphotericin B, which was used as a positive control.

### Cytotoxic Activity of naphthofuranquinone in mammalian V79 cell

Table 2 shows that compounds 1-3 showed cytotoxicity against mammalian V79 cells in comparison to untreated cells (P<0.05), as analyzed by the MTT assay.

**Table 2:** Cytotoxic activity of naphthofuranquinone in mammalian V79 cells.

Mammalian V79 cell CI50 µg/mL-1 (CI 95%)	
Compounds	
1	1,31 (1,18–1,49)
2	1,17 (0,95–1,36)
3	1,49 (1,27–1,61)

Fluconazole was used as a positive control. Data are presented as IC50 values and 95% confidence intervals (95% CI) from three independent experiments performed in triplicate.

## Discussion

The results of this study show that compounds 1-3 have antifungal effects. The antifungal activities of naphthoquinone derivatives against various pathogenic yeasts have been reported in several recent studies [12]. When the strains of *C. tropicalis* were exposed to the naphthofuranquinone compounds, the number of viable cells decreased, indicating impairment of the cell membranes.

*C. tropicalis* cells suffered significant changes in cell size/granularity when exposed to the naphthofuranquinones 1-3. The flow cytometry analysis revealed that the fluconazole-resistant cells showed a decreased size (FSC) and increased granularity (SSC), which are indicative of cell death [33]. In the presence of PI, a proportion of the cells became PI-positive when exposed to compounds 1-3. The increased PI uptake in the fluconazole-resistant *C. tropicalis* cells indicates that these compounds induce cell death, based on the assumption that PI can bind to DNA only by reaching the cores of dead cells [34]. The antifungal effect was most pronounced in the strains treated with compound 3, suggesting a possible structure-activity relationship.

The *C. tropicalis* strains showed an increase in total intracellular levels of ROS after treatment with methylated and iodinated naphthoquinones 1-3. These data corroborate those reported by Xu et al. [34] who demonstrated that ROS levels increased in *Saccharomyces cerevisiae* strains after treatment with a quinone-derived compound. ROS are essential regulators of aging and are reported to be key players in cell death [33]. They also play important roles in the cytotoxicity of quinoidal compounds in various organisms, including yeasts [32]. The above-mentioned increased levels of intracellular ROS can be explained by the action of quinones via the inhibition of cellular respiration enzymes [35].

The mitochondrial function was affected in *C. tropicalis* cells treated with compounds 1-3. The results suggest that these naphthofuranquinones may interfere with the mitochondrial respiratory function, causing the Δψm to collapse and preventing the accumulation of Rho123 in the mitochondria. This evidence corroborates the findings of Emadi et al. [36], who showed that molecules derived from naphthoquinones can promote the depolarization of yeast mitochondrial membranes. A Δψm collapse can lead to transient pore openings in the mitochondrial membranes and the release of pro-apoptotic factors into the cytosol [37]. Cell death in yeast correlates with Δψm dysfunction stemming from oxidative damage caused by ROS accumulation [34]. Therefore, the formation of free radicals appears to be an important mechanism of cytotoxicity caused by the treatment of fluconazole-resistant *C. tropicalis* strains with compounds 1-3.

Cell membranes are often permeable to naphthoquinones, as evidenced by their ability to induce single- or double-strand breaks in DNA molecules [12]. Naphthoquinones are highly lipophilic and as such they are prevented from reaching the intracellular active sites by hydrophobic interactions [10]. The treatment of *C. tropicalis* strains with compounds 1-3 promoted DNA damage, and it is known that structural modifications of nucleotide bases can be a consequence of oxidative stress [30]. Therefore, one can speculate that the naphthoquinoidal compounds 1-3 induced DNA breaks through the generation of intracellular ROS. This result highlights the importance of ROS for the cytotoxic effects of naphthofuranquinones on fluconazole-resistant *C. tropicalis* strains. Indeed, the oxidation of nucleotide bases is as important as DNA breakage for the overall function and survival of the cell [30].

In summary, the present data suggest that these compounds may be used as antifungal agents for the treatment of candidemia.

## Materials and Methods

### Materials

The three naphthofuranquinones used in this study were: compound 1 (2-[iodomethyl]-2,3-dihydronaphtho [2,3-*b*] furan-4,9-dione), compound 2 (2-[iodomethyl]-2,3 dihydrona- phtho[1,2-*b*]furan-4,5-dione) and compound 3 (2-methyl-2,3-dihydronaphtho[1,2-b]furan-4,5-dione), which were synthesized as described by Silva et al. [13]. Compounds 1 and 2 were produced by reacting C-allyl lawsone with metallic iodine in dichloromethane, as shown in Figure 1. Compound 3 was also synthesized from C-allyl lawsone, but a simple cyclization using sulfuric acid was performed (Figure 1). All of the compounds were prepared in yields comparable to those described in the literature [13].

### Isolates

Fourteen strains of FLC-resistant *Candida* spp. isolated from blood, urine and cerebrospinal fluid (CSF) of fungemia patients were used. These strains are part of the Collection of Yeasts of the Laboratory of Bioprospection and Experiments in Yeast (LABEL) affiliated with the School of Pharmacy at the Federal University of Ceará (FF/UFC). The strains were incubated on Sabouraud dextrose agar (Himedia, Mumbai, India) at 37°C for 24 h, and were then cultivated on CHROMagar Candida® medium (Himedia) to assess their purity.

### Molecular identification

Genomic DNA was purified using a CTAB (Cetyl-trimethyl-ammonium-bromide)-based protocol, as previously described [14]. The nuclear DNA region comprising the internal transcribed spacers (ITS1 and ITS2) and the 5.8S rRNA gene was amplified by polymerase chain reaction (PCR) using the primers ITS4 (5′-TCCTCCGCTTATTGATATGC-3′) and ITS5 (5′-GCAAGTAAAAGTCGTAACAAGA-3′), as suggested by White et al. [15]. The PCR products were purified using the GFX PCR DNA and Gel Band Purification Kit (GE Healthcare Life Sciences, Piscataway, NJ, USA). The concentrations of the purified PCR products were determined by measuring the absorbance at 260 nm of ten-fold dilutions [16]. DNA sequencing was performed using the DYEnamic ET terminators cycle sequencing kit (GE Healthcare Life Sciences) according to the manufacturer's protocol, and both strands were sequenced using the ITS4 and ITS5 primers. The sequencing reactions were then analyzed in a MegaBACE 1000 automatic sequencer (GE Healthcare Life Sciences). The sequencing runs were performed as follows: sample injection at 3 kV for 50 s and electrophoresis at 6 kV for 180 min. Automated base calling was performed with Cimarron 3.12 software, and the electropherograms were visualized with Sequence Analyzer v4.0 (Amersham Biosciences, Sunnyvale, CA, USA). The base sequences were deduced by inspection of each processed data trace, and the complete sequences were assembled using the Cap3 software [17]. The resulting sequences were deposited in the GenBank database (accession numbers AB861480-AB861482, AB861486, AB861489-AB861491, KF616834, KF616835, and KF616837-KF616841) and compared to those available in public DNA sequence databases using the BLAST program [18].

### *In vitro* antifungal activities

The broth microdilution (BMD) antifungal susceptibility test was performed according to the document M27-S4 (Clinical and Laboratory Standards Institute, 2012) (CLSI) [19] using RPMI-1640 broth (pH 7.0) buffered with 0.165 M MOPS [3-(N-morpholino) propanesulfonic acid] (Sigma Chemical Co. St Louis, MO, USA). Fluconazole (Sigma Chemical) and naphthofuranquinone (compounds 1-3) were dissolved in distilled water and dimethyl sulfoxide (DMSO; Sigma Chemical), respectively. Fluconazole was tested at a range of concentrations from 0.125–64 µg/mL, whereas the naphthofuranquinones were tested at 0.25–128 µg/mL. The 96-well culture plates were incubated at 35°C for 24 h, and the results were examined visually, as recommended by the CLSI [19]. The MIC was considered to be the concentration that inhibited 50% of fungal growth. The *in vitro* drug interactions were evaluated according to the MIC, and the strains were classified as susceptible (S), or resistant (R). The cutoff points for fluconazole susceptibility were as follows: MIC≤2 µg/mL (S), MIC≥8 µg/mL (R) [20], [21]. The strains *C. parapsilosis* ATCC 22019 and *C. krusei* ATCC 6258 were used as controls [19].

### Cell treatments

To determine their cell density, membrane integrity, mitochondrial transmembrane potential and annexin V staining, fluconazole-resistant strains were exposed to various concentrations (32, 64 and 128 µg/mL) of naphthofuranquinones (compounds 1-3). Fluconazole-susceptible strains were treated with fluconazole (64 µg/mL) at 37°C for 24 h [22]. To evaluate the oxidative stress and DNA damage caused by the treatments, the resistant and susceptible strains of *C. tropicalis* were exposed to varying concentrations (32, 64 and 128 µg/mL) of compounds 1-3 for 24 h. Treatment with amphotericin B (Ampho) (Sigma Chemical) was used as a positive control for cell death; the toxic effects of this substance include the condensation and fragmentation of nuclear chromatin, as well as the accumulation of reactive oxygen species (ROS) [23]. All of the experiments were performed in triplicate in three independent experiments.

### Preparation of yeast suspensions

Cell suspensions were prepared from cultures in the exponential growth phase. The cells were harvested, centrifuged (1,600 *g* for 10 min at 4°C),washed twice with an 0.85% saline solution (1,200 *g* for 5 min at 4°C) and then resuspended (∼10^6^ cells/mL) in HEPES buffer [N-(2-hydroxyethyl) piperazine-N′-(2-ethanesulfonic acid)] (Sigma Chemical) supplemented with 2% glucose, pH 7.2 [21], [22].

### Determination of cell density and membrane integrity

The cell density and membrane integrity of the fungal strains were evaluated by the exclusion of 2 mg/L propidium iodide (PI). Aliquots removed after 24 h incubation with drugs (compounds 1-3; fluconazole; Ampho) were analyzed using flow cytometry. A total of 10,000 events were evaluated per experiment (n = 2), with the cellular debris omitted from the analysis. The cellular fluorescence was then determined using flow cytometry in a Guava EasyCyte Mini System cytometer (Guava Technologies, Hayward, CA, USA) and analyzed with CytoSoft 4.1 software [24].

### Detection of reactive oxygen species (ROS) in yeast

For the detection of ROS produced over a 24 h culture period, cells of *C. tropicalis* sensitive and resistant were incubated with 20 µM CM-H_2_DCFDA [5-(and-6)-chloromethyl-2′, 7′-dichlorodihydrofluorescein diacetate acetyl ester] (Sigma Chemical) for 30 min in the dark at 35°C. Next, the cells were harvested, washed, resuspended in PBS and immediately analyzed using flow cytometry (Guava EasyCyte Mini). CM-H_2_DCFDA readily diffuses through the cell membrane and is hydrolyzed by intracellular esterases to non-fluorescent dichlorofluorescin (DCFH), which is then rapidly oxidized to highly fluorescent DCF (2′,7′-dichlorofluorescein) as a result of a broad range of intracellular oxidative stresses other than H_2_O_2_. The fluorescence intensity of DCF is proportional to the amount of ROS formed inside the cell [25].

### Measurement of mitochondrial transmembrane potential (Δψm)

The mitochondrial transmembrane potential was determined by the retention of rhodamine 123 dye by fungal strains after exposure for 24 h. The cells were washed with phosphate-buffered saline (PBS), incubated with 5 mg/L rhodamine 123 (Rho123) at 37°C for 30 min in the dark, and then washed twice with PBS. Their fluorescence was measured using flow cytometry. A total of 10,000 events were evaluated per experiment (n = 2), and the cellular debris was omitted from the analysis [26].

### Comet assay

The alkaline comet assay was performed essentially as described by [27]. Up to 200 µL of 0.5% agarose (normal-melting point) was spread onto each slide, and this supportive agarose layer was air-dried prior to the application of the cell suspension. Yeast cells were centrifuged in an Eppendorf microcentrifuge for 5 min, washed with distilled water, and resuspended in S-buffer (1 M sorbitol and 25 mM KH_2_PO_4_, pH 6.5). Aliquots of approximately 5×10^4^ cells were mixed with 0.7% low-melting point agarose containing 2 mg/mL zymolyase 20T (Seikagaku Corp., Japan) and were spread over the agarose layer. The slides were then covered with coverslips and incubated for 20 min at 30°C to digest the yeast cell walls and obtain spheroplasts. To minimize the activity of endogenous cellular enzymes, all further procedures were performed at 8–10°C. The coverslips were removed, and the slides were incubated in 30 mM NaOH, 1 M NaCl, 0.1% (m/v) laurylsarcosine and 50 mM EDTA, pH 12.3, for 1 h to lyse the spheroplasts. The slides were washed three times for 20 min each in 30 mM NaOH and 10 mM EDTA, pH 12.4, to unwind the DNA. The slides were then subjected to electrophoresis in the same buffer for 20 min at 0.5 V/cm and 24 mA. After electrophoresis, the slides were neutralized by submerging the slides in 10 mM Tris-HCl at pH 7.5 for 10 min, followed by consecutive 10 min incubations in 76% and 96% ethanol. Finally, the slides were allowed to air-dry and were then stained with ethidium bromide (1 mg/mL) and visualized by fluorescence microscopy.

All of the above steps were conducted in the dark to prevent additional DNA damage [28]. Images of 100 randomly selected cells (50 cells from each of 2 replicate slides) from each experimental group were analyzed. The cells were visually scored and assigned to one of five classes according to tail size (from undamaged, 0, to maximally damaged, 4), and a damage index value was calculated for each sample of cells. The damage index values ranged from 0 (completely undamaged: 100 cells×0) to 400 (displaying maximum damage: 100 cells×4). The ratio of tailed cells (indicating DNA strand breakage) to normal cells was calculated [21], [29].

### Analysis of oxidized DNA purine bases in yeast cells

The levels of oxidized purine bases were estimated with the alkaline comet assay, as described above. Briefly, the slides were removed from the lysis solution and were washed three times in an enzyme buffer (40 mM HEPES, 100 mM KCl, 0.5 mM Na_2_EDTA, and 0.2 mg/mL BSA, pH 8.0), drained, and incubated with 70 µL formamidopyrimidine DNA-glycosylase (FPG) (New England BioLabs, USA) for 30 min at 37°C. Images of 100 randomly selected cells (50 cells from each of 2 replicate slides) from each group were visually analyzed. The number of oxidized purines (FPG-sensitive sites) was then determined by subtracting the number of strand breaks present in samples incubated with buffer alone from the total number of breaks observed after incubation with FPG, according to the methods of da Silva Júnior et al. [30].

### Annexin V staining

Treated and untreated *C. tropicalis* cells were harvested by centrifugation and digested with 2 mg/mL zymolyase 20T (Seikagaku Corp.) in potassium phosphate buffer (PPB, 1 M sorbitol, pH 6.0) for 2 h at 30°C. Protoplasts of *C. tropicalis* were stained with FITC-labeled Annexin V and PI using a FITC-Annexin V apoptosis detection kit (Guava Nexin Kit, Guava Technologies). Subsequently, cells were washed with PPB and incubated in Annexin binding buffer containing 5 µl/ml FITC-Annexin V and 5 µl of PI for 20 min. The cells were then analyzed by flow cytometry (Guava EasyCyte Mini System). For each experiment (n = 3) 10,000 events were evaluated and cell debris was omitted from the analysis [31].

### Cells and cultures

Chinese hamster lung fibroblasts (V79 cells) were kindly provided by Dr. J.A.P. Henriques (Federal University of Rio Grande do Sul, Porto Alegre, Brazil). V79 cells were cultivated under standard conditions in MEM with Earle's salts. All culture media were supplemented with 10% fetal bovine serum, 2 mM glutamine, 100 µg/mL penicillin, and 100 µg/mL streptomycin at 37°C with 5% CO_2_. For evaluation of cytotoxic effects, cells were grown for 2 days prior to treatment with the test substances, and afterwards, the medium was replaced with fresh medium containing the test substance or DMSO solution for control. The final concentration of DMSO in the culture medium was kept constant, less than 0.1% (v/v).

### Inhibition of mammalian V79 cell proliferation – MTT test

Cell growth was quantified by the ability of living cells to reduce the yellow dye (3-(4,5-dimethylthiazol-2-yl)-2,5-diphenyltetrazolium bromide) (MTT, Sigma Chemical) to a purple formazan product. For the experiments, V79 cells were plated in 96-well plates (0.3×10^6^ cells/well), and test compounds (0.039 to 25 µg/mL), dissolved in DMSO (0.1%), were then added to each well, followed by incubation for 72 h. Afterwards, the plates were centrifuged and the medium replaced by fresh medium (150 µL) containing 0.5 mg/mL MTT. Three hours later, the MTT formazan product was dissolved in 150 µL DMSO and absorbance was measured using a multiplate reader (Spectra Count, Packard, Ontario, Canada). The effect of the test substances was quantified as the percentage of control absorbance of the reduced dye at 595 nm. Experiments were carried out in duplicate and repeated at least three times [32].

### Statistical Analysis

The *in vitro* susceptibility experiments were repeated at least three times on different days. Geometric means were used to compare the MIC results. The data obtained from the flow cytometry and alkaline comet assays were compared using a one-way analysis of variance (ANOVA) followed by the Newman-Keuls test (P<0.05).
